# Effects of hatching egg storage duration and warming rate from storage to incubation temperature on morphological broiler embryo development

**DOI:** 10.1016/j.psj.2025.105451

**Published:** 2025-06-17

**Authors:** Anne Pennings, Hendrikus Johannes Wijnen, Carla Willemien van der Pol, Elisabeth Anna Maria Graat, Bas Kemp, Henry van den Brand

**Affiliations:** a Adaptation Physiology Group, Department of Animal Sciences, Wageningen University, De Elst 1, P.O. Box 338, 6700 AH, Wageningen, the Netherlands; b HatchTech Incubation Technology, Innovatielaan 3, 6745 XW, De Klomp, the Netherlands

**Keywords:** Incubation, Egg storage, Warming rate, Embryo development, Hatchability

## Abstract

Hatching egg storage affects the development and survival of broiler embryos. Storage for longer than 7 d is associated with decreased hatchability, delayed hatching, and lower day-old chick quality. These negative effects may be mitigated by the rate at which eggs are warmed from storage temperature (18°C) to incubation temperature (37.8°C), which is referred to as the ‘warming rate’. The current study investigated effects of broiler egg storage duration in interaction with warming rate on morphological embryo development and survival, and how albumen and yolk pH are affected. An experiment with a 2 × 3 factorial arrangement, testing 2 storage durations (4 and 14 d) and 3 warming rates (10, 24, and 144 h), was conducted. During 14 d of storage, embryos advanced morphologically, but during warming and incubation, these embryos lagged behind compared to those stored for 4 d. This might be due to the larger difference between albumen and yolk pH after 14 d of storage, potentially explaining the shorter chicks at hatch compared to a 4 d of storage (19.5 vs 19.6 cm; *P* = 0.04). A 24 h and 144 h warming rate allowed the embryo time to develop before reaching incubation temperature, without affecting growth during incubation. A 144 h warming rate resulted in longer chicks at hatch (19.6 vs 19.5 cm; *P* = 0.04) and a shorter incubation duration (77 h; *P* < 0.01), compared to a 10 h warming rate. After 4 d of storage, hatchability increased from 93.6 % for the 10 h warming rate to 96.0 % following a 144 h warming rate (*P* = 0.04). After 14 d of storage, hatchability was similar for all warming rates. Incubation duration, however, increased for the 10 h and 24 h warming rate compared to 4 d of storage (2 and 9 h respectively; *P* < 0.01), but not for the 144 h warming rate. The absence of a hatch delay suggests that a warming rate of 144 h may have compensated for the developmental delay typically associated with 14 d of egg storage.

## Introduction

The development and survival of broiler embryos during incubation are affected by the duration of pre-incubation egg storage (Fasenko et al., 2001). If, and for how long, hatching eggs are stored before the start of incubation depends on the supply of hatching eggs, hatchery capacity, and the demand for day-old chicks. Hatcheries generally aim to set their eggs after 3 to 5 d of storage to initiate incubation, but storage duration can be extended beyond 7 d (Reijrink et al., 2010a). A storage duration of longer than 7 d is associated with decreased hatchability, delayed hatching, and lower chick quality at hatch (Tona et al., 2003). On average, every day of egg storage until 7 d decreased hatchability with 0.2 %, while a storage duration beyond 7 d decreased hatchability by 0.5 % per day (Yassin et al., 2008). This decrease in hatchability appears to be mainly caused by an increase in embryo mortality in early (d 0-6) and late incubation (d 18-21), while embryo mortality in middle incubation (d 7-17) was not affected (Elibol et al., 2002).

The negative effects of prolonged egg storage on incubation results may be associated with the morphological developmental stage of the embryo, and/or biochemical changes within the egg during storage (Reijrink et al., 2008). Fasenko et al. (2001) suggested that there is an optimal stage of morphological development at which the embryo is best able to resume development after storage. Embryos of Eyal-Giladi and Kochav (1976) (**EGK**) stage XII/XIII were found to be more resistant towards the negative effects of storage than less or more advanced embryos, which was expressed by a lower embryo mortality during incubation (Fasenko et al., 2001). On the one hand, less developed embryos (<EGK stage XII), with fewer and less differentiated cells, may not reach the minimum number of viable embryonic cells required to continue developmental processes once incubation is initiated (Fasenko et al., 1992). On the other hand, storage of more advanced embryos (>EGK stage XIII, i.e. when the formation of the primitive streak begins) also affect embryo viability negatively, probably because this is a period of very active cell migration and differentiation (Bellairs, 1986), and consequently the embryo may not respond well to developmental processes slowing down during storage.

The biochemical changes in egg components, particularly in the albumen and yolk, might play a role in the embryo’s ability to resume development after egg storage. During the first few days of storage, the pH of both the albumen and yolk increases. The pH of the albumen increases from approximately 7.6 at oviposition to approximately 9.5 within 4 d of storage, while the pH of the yolk increases from approximately 6.2 to approximately 6.5 (Freeman and Vince, 1974; Stern, 1991). After 4 d of storage, the difference between the pH of the albumen and yolk is approximately 3 pH units, implying that the albumen is a thousand times more alkaline than the yolk. The relatively large difference between albumen and yolk pH may be suboptimal for the embryo that is situated in between the albumen and yolk and is only separated by the perivitelline membrane. It has been suggested that the large difference in alkalinity between albumen and yolk damages blastoderm cells, leading to the embryo initiating cell death mechanisms, like apoptosis (Arora and Kosin, 1968; Bloom et al., 1998; Hamidu et al., 2011; Pokhrel et al., 2018). The longer eggs are stored, the longer the embryo is exposed to the large difference between albumen and yolk pH, leading to an expected increase in cell death for long-stored eggs. Because of storage-induced cell death, the embryo may lack sufficient healthy blastoderm cells to sustain development, ultimately leading to its death. Therefore, the negative effects of prolonged storage on embryo survival may not only be related to the morphological developmental stage of the embryo, but may also be a result of biochemical changes within the egg.

A potential strategy to mitigate negative effects of prolonged storage on incubation results is related to the rate at which hatching eggs are warmed from storage to incubation temperature, defined as the warming rate (**WR**). Reijrink et al. (2010b) compared 4 h and 24 h of linearly warming 13d-stored eggs from storage temperature (18-20°C) to incubation temperature (37.8°C eggshell temperature; **EST**) and showed that the 24 h WR resulted in a lower embryo mortality during the first 2 days of incubation compared to a WR of 4 h (12.7 % and 17.1 %, respectively), and a higher hatchability (78.9 % and 73.2 %, respectively).

It can be hypothesized that with a slower WR, the gradual increase in temperature facilitates mitosis, increasing the number of cells before incubation begins, resulting in a higher survival probability of the embryo. As cell death is higher in longer stored eggs, the suggested benefit of a slower WR on embryo survival is hypothesized to be larger in 14d-stored eggs than in 4d-stored eggs. However, hardly anything is known about the potential interaction between storage duration and WR, nor about how both factors affect broiler embryo development. The objective of this study was to investigate effects of hatching egg storage duration in interaction with WR on broiler embryo development and survival, and how albumen and yolk pH are affected. We hypothesized that a slower WR advances morphological embryo development pre-incubation, and increases embryo survivability, particularly for embryos from long-stored eggs.

## Materials and methods

### Experimental design

An experiment was set up as a 2 × 3 factorial arrangement with storage duration (4 and 14 d) and WR (10, 24, and 144 h) as treatments. Four consecutive batches were used; in batch 1 and 3, eggs were stored for 4 d, and in batch 2 and 4, eggs were stored for 14 d. In each batch, all three WR treatments were applied. The experiment was executed in 2024 at the hatchery Lagerwey Heijmer van Hulst (Lunteren, the Netherlands), and was approved by the Animal Use and Care committee of Wageningen University and Research; approval number: NAE_2024.W-007.

### Egg storage

A total of 16,200 Ross 308 broiler hatching eggs originating from one single commercial breeder flock (Alphen, the Netherlands, age 37-45 wk) were used in the experiment. Per batch, 4,050 first quality (clean, without cracks) freshly laid eggs were obtained. The day before the experimental eggs were obtained, all eggs were collected just before the lights went out at 1800 h. The next morning, the lights were switched on at 0400 h and experimental eggs were collected in the following 4 h and directly transported on setter trays to the hatchery in a climate-controlled vehicle in which air temperature was kept between 16 and 18°C. Eggs were not disinfected prior to, nor during the experiment. Upon arrival at the hatchery, in each batch, 100 eggs were individually weighed, and the average egg weight and SD were calculated. Additionally, all other eggs were individually weighed and 3,600 eggs (24 trays with 150 eggs) with an average egg weight ± 2 x SD were selected. Eggs were stored on hatching trays with their blunt end up in the egg storage room at the hatchery. During storage, average air temperature was 17.7°C (range: 16.2-22.9°C) and average relative humidity was 77.5 % (range: 41.6-93.5 %). Storage conditions were kept consistent across both storage duration treatments to ensure similarity. Eggs were not turned during storage.

### Warming rate and incubation

**Fig. 1:**
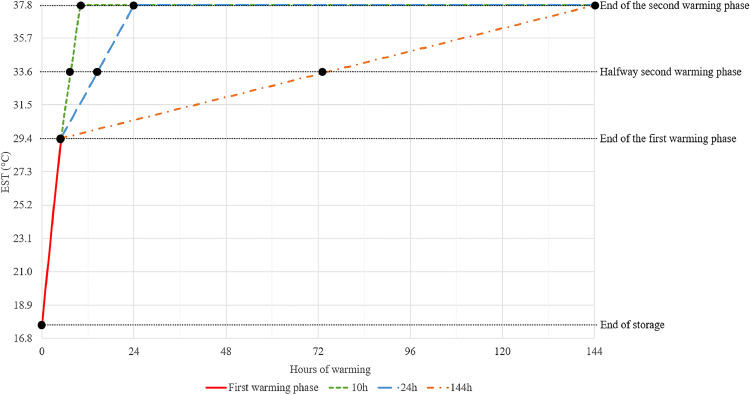
Experimental setup: Warming rate treatments (10 h, 24 h, 144 h) of broiler hatching eggs from storage to incubation temperature with the associated eggshell temperature (**EST**) pattern. Bullets (●) indicate the moments at which albumen and yolk pH were measured, and morphological embryo stage was determined.

Four small-scale setter incubators (PicoClimer, HatchTech Incubation Technology B.V.) with a maximum capacity of 4,800 eggs (one trolley with 32 trays) were used for the experiment. In each batch, three out of these four setters were used. Each WR treatment was assigned to one of the three setters. WR treatments rotated over the four setters between batches. Trays (8/batch per setter) were placed in the middle of the trolley, with empty trays filling the rest of the trolley to guarantee optimal airflow. Once placed in the setters, eggs were warmed from storage air temperature (17.7°C) to EST 37.8°C within 10 h (10 h), 24 h (24 h), or 144 h (144 h). The WR treatments consisted of two phases. In the first phase, all eggs were warmed linearly from 17.7°C to EST 29.4°C in 5 h. The endpoint for the first warming phase (EST 29.4°C) was based on Van Roovert-Reijrink et al. (2018), who showed no effect of the WR (3 to 17 hours) below this EST on early embryo mortality. In the second phase, EST increased linearly from 29.4°C to 37.8°C during the remaining 5, 19, or 139 h for the 10 h, 24 h, and 144 h treatment, respectively (Fig. 1). During warming and incubation, the EST was monitored by four EST sensors (NTC Thermistors: type DC 95; Thermometrics, Somerset, UK) in each setter, which were placed at the equator of the eggshell of four individual eggs, using silicone heat sink compound (Type 340; Dow Corning, Midland, MI) and a small piece (approx. 1.5 × 1.5 cm) of kinesiology tape (Leukotape®K; BSN medical GmbH, Hamburg, Germany). Setter air temperature was continuously and automatically adjusted, based on the median temperature of the four EST sensors to maintain the correct EST. After warming, eggs remained in the same setter and were incubated at a constant EST of 37.8°C throughout the remaining incubation period. Eggs were turned in the setter at an angle of 90° every hour (45° to both sides) during the WR period and incubation until transfer to the hatcher on d 18 of incubation. Relative humidity and CO_2_ levels were monitored throughout the experiment by two separate sensors. During the WR period and the first 3 d of incubation that followed, relative humidity was maintained between 55 and 85 %. Thereafter, relative humidity was lowered to 30 to 40 % throughout the remaining incubation period. During the WR and incubation, the inlet and outlet valve of the setter were controlled to keep CO_2_ levels below 0.35 %.

### Data collection

Eggs from each treatment were collected at various moments to determine albumen and yolk pH, and morphological embryo stage (Fig. 1). The first collection moment took place at the moment of egg arrival at the hatchery, which was on the day of oviposition. Subsequent collecting moments were at the end of storage (4 or 14 d), at the end of the first warming phase (EST 29.4°C), halfway warming from storage to incubation temperature (EST 33.6°C), and at the end of the second warming phase (EST 37.8°C).

At each collection time point, 45 eggs per batch per treatment were randomly selected from the setter. The collected eggs were then transferred to an adjacent lab-scale incubator (Brinsea Ova-Easy Advance 190 EX, Brinsea, Titusville, USA) to maintain an EST comparable to the setter's temperature until measurements were conducted within two hours after collection. During measurement, the eggs were carefully cracked open, and the albumen and yolk were separated. Embryos were isolated from the yolk using the filter ring technique (Gupta and Bakst, 1993). Successfully isolated embryos were staged according to the classification tables of Eyal-Giladi and Kochav (1976) and Hamburger and Hamilton (1951). After removal of the embryo from the yolk, the albumen and yolk were transferred to 50 mL tubes and homogenized on a vortex to determine the pH of the albumen and the yolk with an electrode pH meter (Seven Easy, Mettler Toledo, Schwerzenbach, Switzerland).

Start of incubation (D0) was defined as the first moment that eggs reached an EST of 37.8°C. Embryo weight was measured every three days (**D**) of incubation, so at D3, D6, D9, D12, D15, and D18. The embryos were separated from the egg contents and membranes and were carefully patted dry with paper towels without damaging the embryo. Each embryo was individually weighed to two decimals (Quintix 612-1S, Sartorius GmbH, Göttingen, Germany) and culled thereafter by decapitation. Embryo weight at D3 for the 10 h and 24 h WR treatment is lacking as measurements were unreliable due to too low embryo weight (<0.1 g). During the first batch of the experiment, it was noted that the D15 embryos of the 144 h WR were similar in weight to the D18 embryos of the 10 h and 24 h WR. To prevent chicks from hatching on setter trays, it was decided to transfer from the setter to the hatcher at D15 for the 144 h WR, meaning that embryo weight at D18 for the 144 h WR is also lacking.

On D7 and at transfer (at D18 for the 10 h and 24 h WR, at D15 for the 144 h WR), all eggs were candled manually, and infertile eggs and eggs containing a dead embryo were removed. The removed eggs were cracked open to macroscopically determine infertility and embryo mortality moment. Additionally, at the end of the hatching phase, the unhatched eggs were collected and cracked open to determine the reason and day of death. Total embryo mortality was split up into early mortality (oviposition-D7), middle mortality (D8-D14), and late mortality (D15-hatch). Infertility was calculated as a percentage of the total eggs set minus the number of eggs removed from the setters during the experiment for morphological staging or embryo weight. Embryo mortality and hatchability were calculated as a percentage of the number of fertile eggs set that remained after these corrections.

All setter trays with eggs were weighed on the day of egg arrival, after storage, and on the day of transfer to determine average egg weight and egg weight loss from oviposition until transfer. At transfer, all eggs containing a viable embryo were transferred to hatcher baskets and labelled with the corresponding tray number. The hatcher baskets were positioned on three hatcher trolleys (one trolley per WR treatment) and each trolley was placed in one small-scale hatcher incubator (PicoClimer, HatchTech Incubation Technology B.V.). All hatcher baskets were checked every eight hours to determine the moment of emergence from the eggshell, and chick quality at hatch. During these checks, the total number of hatched chicks was counted to determine hatchability. Chicks were classified as first-grade when it was clean and free of deformities. The remaining chicks were classified as second-grade, including the chicks that died in the hatcher basket after emergence from the eggshell. The proportion of second-grade chicks was expressed as a percentage of the total hatched chicks. The average incubation duration per treatment was calculated by averaging the time in hours it took for each chick to hatch from the start of incubation (moment EST reached EST 37.8°C). From each 30th hatched first-grade chick, body weight and chick length (Hill, 2001) from the tip of the beak to the implantation of the nail of the middle toe were measured (*n* = 350).

### Statistical analyses

Morphological stages were found to largely differ between treatments, meaning that at some measuring points both classification tables (Eyal-Giladi and Kochav, 1976; Hamburger and Hamilton, 1951) needed to be used at the same measuring moment. However, the classification tables are not logically sequential, meaning that the difference in time and developmental processes between successive morphological stages are not equal. Therefore, the results of morphological stage are expressed as averages of the raw data, including a range (minimum stage–maximum stage) reported in the units corresponding to the classification table used.

All other data was analyzed using the statistical software package SAS (version 9.4, SAS Institute 2019) and differences were considered significant when *P* ≤ 0.05. For data collected during storage and the first warming phase (EST ≤29.4°C), the following model was used: Y=μ+Storage+e, where *Y* = the dependent variable, μ = the overall mean, Storage = the storage duration (4 or 14 d), and e = the residual error. For data collected during the second warming phase and incubation (EST >29.4°C), the following model was used: Y=μ+Storage+Warming+Storage*Warming+e, where *Y* = the dependent variable, μ = the overall mean, Storage = the storage duration (4 or 14 d), Warming = the WR (10, 24, or 144 h), Storage*Warming = the interaction between storage duration and WR, and e = the residual error. All values are expressed as least squares means (LSMeans) ± pooled SEM. The LSMeans were compared using the Tukey adjustment for multiple comparisons.

Albumen and yolk pH, embryo weight, and egg weight loss were analyzed with a general linear regression model (Proc GLM), with egg, embryo, and tray as experimental unit, respectively. Homogeneity of variance was tested for both means and residuals. The percentages of infertility, embryo mortality, and hatchability were analyzed with a generalized linear mixed model (Proc GLIMMIX), using a binary distribution and logit link function. Incubation duration, second-grade chicks, chick weight, and chick length were analyzed with a general linear mixed model (Proc MIXED) and homogeneity of variance was tested for both means and residuals. The observers (four persons) were added as a random effect in the analysis of chick length. The statistical analysis of infertility, embryo mortality, hatchability, incubation duration, second-grade chicks, chick weight, and chick length was corrected for the effect of setter tray (*n* = 96).

## Results

### Storage and first warming phase (EST ≤29.4°C)

At the end of storage, just before the start of warming, albumen pH was higher for 14d-stored eggs than for 4d-stored eggs (9.19 vs 9.05; *P* < 0.01; Table 1). At the end of the first warming phase (EST 29.4°C), albumen pH was still higher for 14d-stored eggs than for 4d-stored eggs (9.15 vs 9.06; *P* < 0.01). Yolk pH was 6.2 at the end of storage and at the end of the first warming phase; no effect of storage duration was observed (*P* ≥ 0.17). At oviposition, average morphological stage was EGK 10.3. At the end of storage, embryos from 4d-stored eggs were still EGK stage 10.3, while embryos from 14d-stored eggs advanced to EGK stage 11.3. At the end of the first warming phase, embryos from the 4d-stored eggs were EGK stage 10.5, and embryos from 14d-stored eggs were EGK stage 10.9.

**Table 1:** Albumen and yolk pH of broiler hatching eggs and morphological embryo stage, classified according to Eyal-Giladi and Kochav (1976) (**EGK**), at 1) oviposition, 2) the end of the egg storage period (4d, 14d), and 3) after linearly warming eggs from storage temperature (17.7°C) to an eggshell temperature (**EST**) of 29.4°C in 5 h (first warming phase).

	Albumen pH			Yolk pH			Average morphological embryo stage + (range)		
	Oviposition*	End of storage	End of first warming phase^2^	Oviposition*	End of storage	End of first warming phase	Oviposition*	End of storage*	End of first warming phase*
n^1^	117	97	85	120	95	89	61	64	56
Storage duration									
4d	8.11	9.05^b^	9.06^b^	6.12	6.21	6.20	EGK 10.3 (EGK X–XI)	EGK 10.3 (EGK X–XII)	EGK 10.5 (EGK X–XIII)
14d		9.19^a^	9.15^a^		6.23	6.21		EGK 11.3 (EGK X–XIII)	EGK 10.9 (EGK X–XIII)
SEM	-	0.02	0.02	-	0.01	0.01	-	-	-
P-value	-	<0.01	<0.01	-	0.17	0.34	-	-	-

⁎ Values displayed in these columns are an average of the raw data, no statistical analysis was performed. ^a,b^ Least square means within a column lacking a common superscript differ (*P* ≤ 0.05).

1 Experimental unit in the statistical analysis of albumen and yolk pH was egg (n).

2 The first warming phase refers to linearly warming eggs from 17.7°C to EST 29.4°C and the second warming phase refers to linearly warming eggs from EST 29.4°C to 37.8°C.

### Second warming phase (EST >29.4°C – 37.8°C)

An interaction between storage duration and WR was observed for albumen pH halfway the second warming phase (*P* < 0.01; Table 2). For the 24 h WR, albumen pH was higher for 14d-stored eggs compared to 4d-stored eggs (∆=0.12), whereas for the 10 h and 144 h WR, albumen pH did not differ between storage durations. At the end of the second warming phase, albumen pH was higher for the 24 h WR, compared to the 10 h WR (Δ=0.09; *P* < 0.01), and could not be measured for the 144 h WR due to the advanced morphological stage of the embryo.

**Table 2:** Albumen and yolk pH of broiler hatching eggs and morphological embryo stage, classified according to Eyal-Giladi and Kochav (1976) (**EGK**) or Hamburger and Hamilton (1951) (**HH**), after two storage durations (4d, 14d) at 1) halfway warming at an eggshell temperature (**EST**) of 33.6°C (second warming phase), and 2) at the end of warming eggs linearly from EST 29.4°C to 37.8°C in 5 h (10 h), 19 h (24 h), or 139 h (144 h).

	Albumen pH		Yolk pH		Average morphological embryo stage + (range)	
	Halfway second warming phase^2^	End of warming	Halfway second warming phase	End of warming	Halfway second warming phase*	End of warming*
n^1^	301	273	291	268	173	174
Storage duration						
4d	9.17	9.20	6.22^b^	6.19^b^	HH 2.5 (EGK X–HH 9-)	HH 9.6 (EGK X–HH 21)
14d	9.19	9.21	6.27^a^	6.26^a^	EGK 14.7 (EGK X–HH 8)	HH 8.5 (EGK XI–HH 20)
SEM	0.01	0.01	0.01	0.01	-	-
Warming rate						
10h	9.11	9.16^b^	6.21^b^	6.22	EGK 11.6 (EGK X–XV)	EGK 12.8 (EGK X–XV)
24h	9.14	9.25^a^	6.23^b^	6.22	EGK 12.8 (EGK XI–XIV)	EGK 14.2 (EGK XIII–HH 4)
144h	9.30	NA	6.29^a^	NA	HH 7.6 (HH 6–9-)	HH 19.8 (HH 19–21)
SEM	0.01	0.01	0.01	0.01	-	-
Storage duration x Warming rate						
4d x 10h	9.12^c^	9.16	6.19	6.18	EGK 10.9 (EGK X–XIII)	EGK 12.8 (EGK X–XIV)
4d x 24h	9.08^c^	9.23	6.20	6.19	EGK 13.0 (EGK XII–XIV)	EGK 14.5 (EGK XIII–HH 4)
4d x 144h	9.32^a^	NA	6.25	NA	HH 7.5 (HH 6–9-)	HH 20.2 (HH 19–21)
14d x 10h	9.09^c^	9.15	6.23	6.26	EGK 12.2 (EGK X–XV)	EGK 12.8 (EGK XI–XV)
14d x 24h	9.20^b^	9.27	6.25	6.26	EGK 12.7 (EGK XI–XIV)	EGK 14.0 (EGK XIII–HH 3)
14d x 144h	9.29^a^	NA	6.33	NA	HH 6.4 (HH 6–8)	HH 19.3 (HH 19–20)
SEM	0.02	0.01	0.01	0.01	-	-
P-values						
Storage duration	0.15	0.31	<0.01	<0.01	-	-
Warming rate	<0.01	<0.01	<0.01	0.79	-	-
Storage duration x Warming rate	<0.01	0.06	0.27	0.85	-	-

⁎ Values displayed in these columns are an average of the raw data, no statistical analysis was performed.

a-c Least square means within a column and treatment lacking a common superscript differ (*P* ≤ 0.05).

1 Experimental unit in the statistical analysis of albumen and yolk pH was egg (n).

2 The first warming phase refers to linearly warming eggs from 17.7°C to EST 29.4°C and the second warming phase refers to linearly warming eggs from EST 33.6°C to 37.8°C. NA = Not Available: Albumen and yolk pH of the 144 h treatment could not be measured at the end of the second warming phase due to the advanced morphological stage of the embryo.

No interaction between storage duration and WR was observed for yolk pH (*P* > 0.27; Table 2). Halfway the second warming phase and at the end of warming, yolk pH was higher (Δ=0.07 and Δ=0.05, respectively) for 14d-stored eggs compared to 4d-stored eggs (both *P* < 0.01). Halfway the second warming phase, yolk pH was higher for the 144 h WR compared to the 10 h and 24 h WR (Δ=0.08 and Δ=0.06, respectively; *P* < 0.01), which did not differ from each other.

Halfway the second warming phase and at the end of warming, embryos from 4d-stored eggs were numerically more advanced (HH stage 2.5 and 9.6, respectively) than embryos from 14d-stored eggs (EGK stage 14.7 and HH stage 8.5, respectively). Halfway the second warming phase and at the end of warming, embryos from the 144 h were most advanced (HH stage 7.6 and 19.8, respectively), embryos from the 10 h WR were the least advanced (EGK stage 11.6 and 12.8, respectively), and embryos from the 24 h WR were intermediate (EGK stage 12.8 and 14.2, respectively).

### Incubation (EST 37.8°C)

An interaction between storage duration and WR was observed for embryo weight at D12 and D18 (*P* ≤ 0.03; Table 3). At D12, embryos from 4d-stored eggs were heavier than embryos from 14d-stored eggs for all WR treatments, but the difference was most profound for the 24 h WR (Δ=0.66 g, Δ=1.51 g, and Δ=0.80 g, for the 10 h, 24 h, and 144 h WR, respectively). At D18, embryos from the 4d-stored eggs did not differ in weight between the 10 h and 24 h WR, while for the 14d-stored eggs, embryos from the 24 h WR were 1.78 g heavier than embryos from the 10 h WR. At D3, D6, D9, and D15, embryos from the 4d-stored eggs were heavier than embryos from the 14d-stored eggs regardless of WR (Δ=0.08, Δ=0.11 g, Δ=0.26 g, and Δ=0.73 g, respectively; all *P* < 0.01). At D6, D9, and D15, embryo weight of all WR treatments differed from each other, and increased as the WR became progressively more gradual (all *P* < 0.01).

**Table 3:** Weight of broiler embryos measured every three days of incubation (D), affected by storage duration (4d, 14d) and warming rate from storage to incubation temperature (10 h, 24 h, 144 h).

	Embryo weight (g)					
	D3	D6	D9	D12	D15	D18
n^1^	60	180	180	179	180	120
Storage duration						
4d	0.61^a^	1.20^a^	4.11^a^	11.73	23.30^a^	31.77
14d	0.53^b^	1.09^b^	3.85^b^	10.74	22.57^b^	31.31
SEM	0.01	0.01	0.03	0.09	0.14	0.28
Warming rate						
10h	NA	0.44^c^	2.04^c^	6.77	18.01^c^	31.08
24h	NA	0.60^b^	2.30^b^	8.21	18.96^b^	32.00
144h	0.57	2.39^a^	7.60^a^	18.72	31.84^a^	NA
SEM	-	0.01	0.04	0.11	0.17	0.28
Storage duration x Warming rate						
4d x 10h	NA	0.47	2.10	7.10^d^	18.54	31.73^ab^
4d x 24h	NA	0.64	2.46	8.96^c^	19.46	31.81^ab^
4d x 144h	0.61	2.47	7.79	19.12^a^	31.91	NA
14d x 10h	NA	0.40	1.98	6.44^e^	17.48	30.42^b^
14d x 24h	NA	0.55	2.15	7.45^d^	18.47	32.20^a^
14d x 144h	0.53	2.32	7.41	18.32^b^	31.77	NA
SEM	-	0.02	0.06	0.16	0.23	0.40
P-values						
Storage duration	<0.01	<0.01	<0.01	<0.01	<0.01	0.25
Warming rate	-	<0.01	<0.01	<0.01	<0.01	0.02
Storage duration x Warming rate	-	0.10	0.07	0.02	0.10	0.03

a-e Least square means within a column and treatment lacking a common superscript differ (*P* ≤ 0.05).

1 Experimental unit in the statistical analysis of embryo weight was embryo (n). NA = Not Available: Embryo weight was not measured at D3 for the 10 h and 24 h treatments due to the small size of the embryo, and embryo weight was not measured at D18 for the 144 h treatment due to transfer from setter to hatcher taking place on D15.

**Fig. 2:**
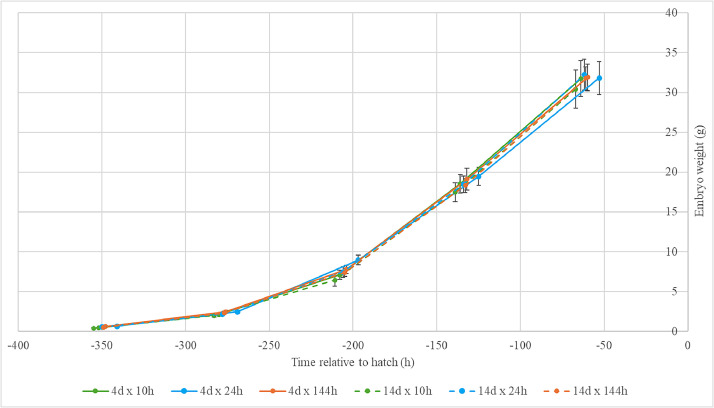
Broiler embryo weight (g) excluding the yolk sac at various moments during incubation (h relative to average hatch moment per treatment) after two storage durations (4d, 14d) and three warming rates from storage to incubation temperature (10 h, 24 h, 144 h). Error bars indicate the SEM.

Embryo weight was measured every 3 d of incubation, but due to differences in embryo development among treatment groups, the moments at which embryo weight was measured did not correspond to the same embryonic developmental stage. To facilitate a sound comparison, embryo weight during incubation was related to hatch moment per treatment group (storage duration x WR) (Fig. 2). The embryo weight curve was found to be similar for all treatment groups, though this could not be statistically analyzed.

### Oviposition until hatch

An interaction between storage duration and WR was observed for egg weight loss from oviposition to transfer to the hatcher (*P* < 0.01; Table 4). For the 24 h WR, egg weight loss did not differ between storage durations, but for the 10 h and 144 h WR, egg weight loss was higher for the 14d-stored eggs than for the 4d-stored eggs (Δ=2.3 % and Δ=1.3 %, respectively).

**Table 4:** Broiler egg weight loss from oviposition until transfer, infertility (% of total eggs set), and early- (oviposition-D7), middle- (D8-14), late- (D15-hatch), and total (oviposition-hatch) embryo mortality, and hatchability (% of fertile eggs set) affected by storage duration (4d, 14d) and warming rate from storage to incubation temperature (10 h, 24 h, 144 h).

	Egg weight loss (%)	Infertility (%)	Embryo mortality				Hatchability (%)
			Early (%)	Middle (%)	Late (%)	Total (%)	
n^1^	96	12,010	11,552	11,552	11,552	11,552	11,552
Storage duration							
4d	11.1	2.8	2.9	1.0	1.1	5.1	94.9
14d	12.2	3.1	4.9	0.9	1.5	7.4	92.6
SEM	0.1	0.2	0.3	0.1	0.2	0.3	0.3
Warming rate							
10h	11.9	2.8	4.4	1.1	1.3	6.9	93.1
24h	11.7	2.8	3.6	0.8	1.4	6.0	94.0
144h	11.2	3.3	3.4	1.0	1.1	5.6	94.4
SEM	0.1	0.3	0.3	0.2	0.2	0.4	0.4
Storage duration x Warming rate							
4d x 10h	10.7^c^	2.4	4.1^a^	0.9^ab^	1.4	6.4^ab^	93.6^bc^
4d x 24h	11.9^b^	2.9	2.6^b^	1.3^a^	1.1	5.0^bc^	95.0^ab^
4d x 144h	10.6^c^	3.4	2.3^b^	0.9^ab^	0.8	4.0^c^	96.0^a^
14d x 10h	13.0^a^	3.3	4.8^a^	1.4^a^	1.3	7.5^a^	92.5^c^
14d x 24h	11.6^b^	2.7	4.8^a^	0.5^bc^	1.7	7.0^a^	93.0^c^
14d x 144h	11.9^b^	3.3	5.0^a^	1.1^ab^	1.5	7.6^a^	92.4^c^
SEM	0.2	0.4	0.5	0.2	0.3	0.6	0.6
P-values							
Storage duration	<0.01	0.43	<0.01	0.59	0.07	<0.01	<0.01
Warming rate	<0.01	0.38	0.05	0.55	0.65	0.06	0.06
Storage duration x Warming rate	<0.01	0.31	0.03	0.02	0.20	0.04	0.04

a-c Least square means within a column and treatment lacking a common superscript differ (*P* ≤ 0.05).

1 The statistical analysis of infertility, embryo mortality, and hatchability was corrected for the effect of setter tray (*n* = 16/interaction treatment group).

The percentage of infertile eggs was ∼3 % and did not differ between treatments groups (*P* > 0.31; Table 4). An interaction between storage duration and WR was observed for early, middle, and total embryo mortality (*P* ≤ 0.04; Table 4). The 24 h and 144 h WR, for 4d-stored eggs, had a lower early embryo mortality than the 10 h WR for 4d-stored eggs and all WR treatments for 14d-stored eggs, which did not differ from each other. The 24 h WR, for 14d-stored eggs, had a lower middle embryo mortality than the 24 h WR for 4d-stored eggs and the 10 h WR for 14d-stored eggs, which did not differ from each other and from all other treatments. The 144 h WR, for 4d-stored eggs, had a lower total embryo mortality than the 10 h WR for 4d-stored eggs and all WR treatments for 14d-stored eggs, while being similar to the 24 h WR for 4d-stored eggs. The 24 h WR, for 4d-stored eggs, had a lower total embryo mortality than all WR treatments for 14d-stored eggs, which did not differ from each other. As hatchability is the reciprocal complement of total embryo mortality, the effects are equivalent. Late embryo mortality was not affected by storage duration, WR, and their interaction (*P* > 0.07).

For incubation duration, an interaction between storage duration and WR was observed (*P* < 0.01; Table 5). For the 10 h and 24 h WR, incubation took longer for 14d-stored eggs compared to 4d-stored eggs (∆=2 h and ∆=9 h, respectively), whereas for the 144 h WR, incubation duration did not differ between storage durations.

**Table 5:** Incubation duration (h at an eggshell temperature of 37.8°C), second-grade broiler chicks (% of total chicks hatched), and chick quality at hatch (chick weight, chick length), affected by storage duration (4d, 14d) and warming rate from storage to incubation temperature (10 h, 24 h, 144 h).

	Incubation duration (h)	Second-grade chicks (%)	Chick weight at hatch (g)	Chick length (cm)
n^1^	10,828	10,828	350	350
Storage duration				
4d	468	0.5	48.1	19.6^a^
14d	472	0.2	48.0	19.5^b^
SEM	0.2	0.1	0.3	0.1
Warming rate				
10h	498	0.3	48.0	19.5^ab^
24h	490	0.4	47.7	19.5^b^
144h	421	0.3	48.6	19.6^a^
SEM	0.2	0.1	0.3	0.1
Storage duration x Warming rate				
4d x 10h	497^b^	0.4	48.5	19.6
4d x 24h	486^d^	0.4	47.2	19.6
4d x 144h	421^e^	0.6	48.6	19.6
14d x 10h	499^a^	0.2	47.5	19.4
14d x 24h	495^c^	0.3	48.2	19.4
14d x 144h	421^e^	0.2	48.5	19.6
SEM	0.3	0.1	0.5	0.1
P-values				
Storage duration	<0.01	0.054	0.89	0.04
Warming rate	<0.01	0.89	0.20	0.04
Storage duration x Warming rate	<0.01	0.35	0.11	0.17

a-e Least square means within a column and treatment lacking a common superscript differ (*P* ≤ 0.05).

1 The statistical analysis of incubation duration, second-grade chicks, chick weight at hatch, and chick length was corrected for the effect of setter tray (*n* = 16/interaction treatment group).

The percentage of second-grade chicks (<1 %, *P* ≥ 0.05; Table 5) and chick weight at hatch (∼48 g, *P* ≥ 0.11) were not different between storage duration, WR, and their interaction. No interaction between storage duration and WR was observed for chick length (*P* = 0.17). Chicks from 4d-stored eggs were 0.1 cm longer than chicks from 14d-stored eggs (*P* = 0.04). Chicks from the 144 h WR were 0.1 cm longer than chicks from the 10 h and 24 h WR, however only the latter significantly different (*P* = 0.04).

## Discussion

The objective of this study was to investigate effects of storage duration in interaction with WR on broiler embryo development and survival, and how albumen and yolk pH are affected. In this discussion, first the main effects of storage duration will be discussed, followed by the main effects of WR, and finally, the interaction effects between storage duration and WR. This structure reflects the biological order of events, as the embryo's condition at the end of storage determines its initial state before a WR treatment is applied, and allows for the independent effects to be understood before exploring their combined influence.

### Storage duration

Morphological development was found to occur during storage of hatching eggs at 17.7°C (range: 16.2-22.9°C), with embryos progressing from EGK stage 10.3 at oviposition to EGK stage 11.3 after 14 d of storage, while remaining at a similar stage after 4 d of storage. This is in accordance with Pokhrel et al. (2018), who found that embryos advanced from EGK stage 10.9 at oviposition to EGK stage 12.4 after 14 d of storage at 18°C. The temperature at which morphological embryo development is halted has been termed the “physiological zero” (Edwards, 1902) and was reported to be approx. 14°C (Fasenko et al., 1992). As storage temperature exceeded this “physiological zero” in the current study, morphological development was expected during 14 d of storage. Even though storage temperature was similar during 4 d of storage, embryos were not found to advance morphologically during this time. So, it appears that morphological embryo development is affected by the combined effects of storage temperature and storage duration.

Even though embryos from 14d-stored eggs were numerically more advanced than embryos from 4d-stored eggs after storage, they quickly started to lag behind in development during warming. Already halfway warming (EST 33.6°C), embryos from 4d-stored eggs were numerically more advanced (HH stage 2.5) than embryos from 14d-stored eggs (EGK stage 14.7). This advanced development continued during incubation. 3 d after the warming phase ended, embryos from 4d-stored eggs were 15 % heavier than embryos from 14d-stored eggs. This suggests that, although embryos were further developed after 14 d of storage, once warming starts, they rapidly lag behind compared to embryos from 4d-stored eggs, suggesting that morphological stage at the end of storage is not the only (and perhaps not the best) indicator for development during incubation.

The reason that the morphological development of embryos from 14d-stored eggs is overtaken by that of 4d-stored eggs during warming might be linked to changes in the egg microenvironment that occur during storage. In the current study, yolk pH was 6.12 at oviposition and increased to 6.21 and 6.23 after 4 d and 14 d of storage, respectively. Simultaneously, albumen pH was 8.11 at oviposition and increased to 9.05 and 9.19 after 4 d and 14 d of storage, respectively. The longer exposure to the quite large pH difference between albumen and yolk during 14 d of storage may be suboptimal for the embryo that is situated in between the albumen and yolk and is only separated by the perivitelline membrane. Prolonged exposure of the embryo to a large albumen-to-yolk pH difference increases the risk of embryonic cell damage. Cell damage is suggested to trigger excessive cell death, which has previously been reported after prolonged storage durations (Bloom and Muscarella, 1998; Hamidu et al., 2011; Pokhrel et al., 2018). Increased cell death during storage leaves the embryo with less viable cells, which is suggested to hinder the continuation of development and growth after prolonged storage.

This raises the question of how morphological development can occur during 14 d of storage, despite a reported increase in cell death during prolonged storage (Bloom and Muscarella, 1998; Hamidu et al., 2011; Pokhrel et al., 2018). It should be noted that mitosis also occurs at temperatures as low as 7.2°C (Arora and Kosin, 1968), increasing the number of embryonic cells during storage. It can be speculated that the balance between cell division and cell death plays a crucial role in determining whether the embryo continues to develop.

In the current study, chick weight at hatch was not affected by storage duration, aligning with Tona et al. (2004) and Reijrink et al. (2008, 2010b). Chick weight included the residual yolk (**RY**) sac, for which the amount can vary between 0.8-10.6 g (Wolanski et al., 2006). Therefore, chick weight, when the RY is included, does not accurately reflect the amount of nutrients utilized for growth during incubation. Chick length at hatch was found to be positively correlated (*r* = 0.60) to the amount of RY, indicating that longer chicks had used more RY to put towards growth and development during incubation (Wolanski et al., 2006). In the current study, chicks from 14d-stored eggs were found to be on average 0.1 cm shorter at hatch than chicks from 4d-stored eggs, which is equal to results from Reijrink et al. (2010b). It can be suggested that the lagging behind of embryo development after prolonged storage led to a suboptimal nutrient utilization, resulting in shorter chicks, of a similar weight, at hatch.

### Warming rate

It can be hypothesized that there is a critical period in embryo development during which embryos particularly benefit from a slower WR. It was suggested that embryos of >EGK stage XIII particularly start to benefit from a slow WR (Van Roovert-Reijrink et al., 2018), as the formation of the primitive streak begins, which involves active cell migration and differentiation (Bellairs, 1986). In support of this, the authors found that embryos did not advance beyond EGK stage XIII when EST≤29.4°C, and the rate of warming (3, 8, 13, or 16 h, linear warming) below this temperature had no effect on early embryo mortality (D0-D7). Similarly, in the current study, none of the embryos exceeded EGK stage XIII when an EST of 29.4°C was reached. This suggests that the critical period during which embryos benefit from a slower WR occurs after the first warming phase, when EST exceeds 29.4°C.

A slower WR >EST 29.4°C allowed the embryo more time to develop before incubation temperature (EST 37.8°C) was reached, resulting in more advanced embryos at the end of warming. At the end of warming/start of incubation, embryos from the 144 h WR were found to be on average HH stage 19.8. According to Hamburger and Hamilton (1951), embryos reached HH stage 20 after approx. 70 to 72 h of incubation, which was then defined as the time spent at an air temperature of 39.4°C. So, even though warming took a total of 6 d in case of the 144 h WR treatment, at the end of warming embryos were found to be similar to an embryo age of approx. 3 d when exposed to a general WR. Average incubation duration (time spent at 37.8°C till hatch) was found to decrease with approx. 3 d (77 h) as well for chicks from the 144 h WR, compared to chicks from the 10 h WR. Even though a slower WR allows the embryo time to develop morphologically before reaching incubation temperature, the embryo growth curve during incubation was similar for all treatments when plotted against time till hatch.

No effect of WR on chick weight at hatch was found in the current study. This was expected as chick weights did not differ between 4 and 24 h of linear warming (Reijrink et al., 2010b). Additionally, the authors found no effect of a WR of 24 h on chick length at hatch, which was then the slowest WR reported in literature. In the current study, however, chicks from the 144 h WR were 0.1 cm longer than chicks from the 10 h and 24 h WR, however only the latter significantly different. A slower WR prolongs the time to reach incubation temperature, extending the period between EST 29.4°C and 37.8°C, and thus extending the period during which embryo development will take place. Already below EST 37.8°C, mitosis occurs (Arora and Kosin, 1968; Konishi and Kosin, 1974; Pokhrel et al., 2018), implying that the number of viable cells increases. This may allow the embryo to recover from cell death that occurred during storage (Bloom and Muscarella, 1998; Hamidu et al., 2011; Pokhrel et al., 2018). It can be hypothesized that a 144 h WR resulted in more advanced embryos with more cells at the start of incubation, enabling unhindered continuation of development and growth, resulting in longer chicks at hatch. A higher chick length at hatch is positively correlated to yolk-free body mass at hatch and growth performance during the rearing period (Molenaar et al., 2007; Wolanski et al., 2004, 2006) while being negatively correlated to first week mortality (Hill, 2001). Therefore, it can be suggested that following a 144 h WR, the embryo may use more residual yolk for development during the incubation period, resulting in better later-life growth and survival.

### Interaction between storage duration and warming rate

It was hypothesized that a slower WR facilitates pre-incubation recovery of cell death that occurred during storage. Consequently, a slower WR was expected to improve embryo survival during incubation, especially after prolonged storage durations. Hatchability was 2.4 % higher for the 144 h WR than for the 10 h WR, but not different from the 24 h WR, after 4 d of storage. After 14 d of storage, however, hatchability was similar for all WR treatments. This is opposite to the findings of Reijrink et al. (2010b), who found no effect of WR (4 and 24 h, linear warming) on hatchability after 4 d of storage. However, after 13 d of storage, the authors found that hatchability was 5.7 % higher when eggs were linearly warmed for 24 h compared to 4 h, mainly expressed by a lower embryo mortality (4.4 %) during the first 2 d of incubation. Embryo survival in general is influenced by the age of the breeder flock age (Mather and Laughlin, 1979) and breeder strain (Yoo and Wientjes, 1991), but may also be affected by the overall laying and fertility performance of the breeder flock. In the current study, hatchability was 94.9 % and 92.6 % for the 4d- and 14d-stored eggs respectively. This is remarkably high, especially after 14 d of storage, as after 13 d of storage, hatchability was found to be between 73.2 % and 78.9 % (Reijrink et al., 2010b). The current study used eggs from a prime breeder flock (37-45 wk), while the study of Reijrink et al. (2010b) used eggs from a young breeder flock (28-29 wk). It can be speculated that eggs from a prime flock with an overall high level of reproductive performance, as in the current study, are more resistant towards the negative effects of prolonged storage, compared to eggs from young and old flocks, with lower overall reproductive performance. However, this does not explain the effect of WR on the hatchability of 4d-stored eggs in the current study. Further research is necessary to investigate physiological processes that might play a role in the interaction between storage duration, WR, and breeder flock characteristics.

Increased cell death during storage may leave the embryo with too few viable cells to sustain development once incubation begins (Fasenko, 2007; Reijrink et al., 2008), resulting in higher early embryo mortality or delayed embryo development. The latter might result in lower embryo weights during incubation (Christensen et al., 2002; Hamidu et al., 2011). In the current study, an interaction between storage duration and WR was found at D12 and D18, while similar patterns, yet insignificant (0.07≤*P* ≤ 0.10), were found at D6, D9, and D15. In general, embryos from 4d-stored eggs were heavier than embryos from 14d-stored eggs up until D15 for the 10 h and 24 h WR, and up until D12 for the 144 h WR. Interestingly, at D18 for the 10 h and 24 h WR, and at D15 for the 144 h WR, embryo weight was similar for embryos from 4d- and 14d-stored eggs. So, it seems that up until the second week of incubation, embryo weight is negatively influenced by storage duration, while this is no longer the case near the end of the incubation period.

Prolonged storage has been reported to be associated with delayed hatching, which was suggested to be the result of a delay in the initiation of embryo development once incubation starts (Becker et al., 1968; MacLaury and Insko, 1968; Mather and Laughlin, 1976; Fasenko and Robinson, 1998; Tona et al., 2003; Fasenko, 2007). For the 10 h and 24 h WR, average incubation duration was indeed 2 and 9 h longer for the 14d-stored eggs than for the 4d-stored eggs, respectively. However, for the 144 h WR, average incubation duration was not affected by storage duration, meaning that chicks from 4d- and 14-stored eggs hatched at the same time. An explanation for this may be that 144 h of warming resulted in more morphologically developed embryos with possibly more cells at the start of incubation (EST 37.8°C), enabling the embryo to make a “head start” at the moment incubation temperature was reached. This “head start” for embryos from 14d-stored eggs may have lasted all the way until hatch, explaining the absence of a hatch delay for chicks following a 144 h WR.

It can be concluded that 14 d of hatching egg storage led to a more morphologically advanced embryo at the end of storage, yet their development progressed more slowly during warming from storage to incubation temperature and early incubation, which is possibly due to a larger difference between albumen and yolk pH in long-stored eggs. A slower warming rate from EST 29.4°C to 37.8°C allows the embryo to (morphologically) develop before reaching incubation temperature, which may improve the ability of the embryo to continue development. The absence of a hatch delay suggests that a warming rate of 144 h may have compensated for the developmental delay typically associated with prolonged egg storage. A warming rate of 24 and 144 h increased hatchability after 4 d of storage, demonstrating the potential of a slower warming rate to improve embryo survival. However, a slower warming rate did not affect hatchability after 14 d of storage, highlighting the need to investigate the underlying physiological mechanisms.

## Declaration of competing interest

The authors declare the following financial interests/personal relationships which may be considered as potential competing interests:

Anne Pennings reports financial support was provided by HatchTech BV. If there are other authors, they declare that they have no known competing financial interests or personal relationships that could have appeared to influence the work reported in this paper.

## References

[bib0001] Arora K.L., Kosin I.L. (1968). The response of the early chicken embryo to pre-incubation temperature as evidenced from its gross morphology and mitotic pattern. Physiol. Zool..

[bib0002] Becker W.A., Spencer J.V., Swartwood J.L. (1968). Carbon dioxide during storage of chicken and turkey hatching eggs. Poult. Sci..

[bib0003] Bellairs R. (1986). The primitive streak. Anat. Embryol..

[bib0004] Bloom S.E., Muscarella D.E. (1998). Stress responses in the avian early embryo: regulation by pro- and anti-apoptotic cell death genes. Poult. Avian Biol..

[bib0005] Bloom S.E., Muscarella D.E., Lee M.Y., Rachlinski M. (1998). Cell death in the avian blastoderm: resistance to stress-induced apoptosis and expression of anti-apoptotic genes. Cell Death Differ..

[bib0006] Christensen V.L., Wineland M.J., Fasenko G.M., Donaldson W.E. (2002). Egg storage alters weight of supply and demand organs of broiler chicken embryos. Poult. Sci..

[bib0007] Edwards C.L. (1902). The physiological zero and the index of development for the egg of the domestic fowl, Gallus Domesticus. Am. J. Physiol..

[bib0008] Elibol O., Peak S., Brake J. (2002). Effect of flock age, length of egg storage, and frequency of turning during storage on hatchability of broiler hatching eggs. Poult. Sci..

[bib0009] Eyal-Giladi H., Kochav S. (1976). From cleavage to primitive streak formation: a complementary normal table and a new look at the first stages of the development of the chick: I. General morphology. J. Dev. Biol..

[bib0010] Fasenko G.M., Robinson F.E., Harden R.T., Wilson J.L. (1992). Variability in preincubation embryonic development in domestic fowl. 2. Effects of duration of egg storage period. Poult. Sci..

[bib0011] Fasenko G.M., Robinson F.E. (1998). Identification of the incubation period when broiler breeder embryonic development is delayed due to egg storage for 14 versus 4 days. Poult. Sci..

[bib0012] Fasenko G.M., Robinson F.E., Whelan A.I., Kremeniuk K.M., Walker J.A. (2001). Prestorage incubation of long-term stored broiler breeder eggs: 1. effects on hatchability. Poult. Sci..

[bib0013] Fasenko G.M. (2007). Egg storage effects on plasma glucose and supply and demand tissue glycogen concentrations of broiler embryos. Poult. Sci..

[bib0014] Freeman B.M., Vince M.A. (1974).

[bib0015] Gupta S.K., Bakst M.R. (1993). Turkey embryo staging from cleavage through hypoblast formation. J. Morphol..

[bib0016] Hamburger V., Hamilton H.L. (1951). A series of normal stages in the development of the chick embryo. J. Morphol..

[bib0017] Hamidu J.A., Uddin Z., Li M., Fasenko G.M., Guan L.L., Barreda D.R. (2011). Broiler egg storage induces cell death and influences embryo quality. Poult. Sci..

[bib0018] Hill D. (2001). Chick length uniformity profiles as a field measurement of chick quality. Avian Poult. Biol. Rev..

[bib0019] Konishi T., Kosin I.L. (1974). Morbidity of aging non-incubated chicken blastoderms: further cytological evidence and interpretation. J. Embryol. Exp. Morphol..

[bib0020] MacLaury D.W., Insko W.M. (1968). Relation of pre-incubation factors and post-hatching performance to length of incubation period: 1. Effects of egg weight and storage time on length of incubation period. Poult. Sci..

[bib0021] Mather M.C., Laughlin K.F. (1976). Storage of hatching eggs: the effect on total incubation period. Br. Poult. Sci..

[bib0022] Mather C.M., Laughlin K.F. (1979). Storage of hatching eggs: the interaction between parental age and early embryonic development. Br. Poult. Sci..

[bib0023] Molenaar, R., Reijrink I.A.M., Meijerhof R., and van den Brand H.. 2007. Relationship between chick length and chick weight at hatch and slaughter weight and breast meat yield in broilers [abstract].

[bib0024] Pokhrel N., Ben-Tal Cohen E., Genin O., Ruzal M., Sela-Donenfeld D., Cinnamon Y. (2018). Effects of storage conditions on hatchability, embryonic survival and cytoarchitectural properties in broiler from young and old flocks. Poult. Sci..

[bib0025] Reijrink I.A.M., Meijerhof R., Kemp B., van den Brand H. (2008). The chicken embryo and its micro environment during egg storage and early incubation. World’s Poult. Sci. J..

[bib0026] Reijrink I.A.M., Meijerhof R., Kemp B., van den Brand H. (2010). Influence of egg warming during storage and hypercapnic incubation on egg characteristics, embryonic development, hatchability, and chick quality. Poult. Sci..

[bib0027] Reijrink I.A.M., Berghmans D., Meijerhof R., Kemp B., van den Brand H. (2010). Influence of egg storage time and preincubation warming profile on embryonic development, hatchability, and chick quality. Poult. Sci..

[bib0028] Stern, C.D. 1.991. The sub-embryonic fluid of the egg of the domestic fowl and its relationship to the early development of the embryo. Pages 81–90 in Avian Incubation. Butterworth-Heinemann, ed. S. G. Tullett, London, UK.

[bib0029] Tona K., Bamelis F., De Ketelaere B., Bruggeman V., Moraes V., Buyse J., Onagbesan O., Decuypere E. (2003). Effects of egg storage time on spread of hatch, chick quality, and chick juvenile growth. Poult. Sci..

[bib0030] Tona K., Onagbesan O., De Ketelaere B., Decuypere E., Bruggeman V. (2004). Effects of age of broiler breeders and egg storage on egg quality, hatchability, chick quality, chick weight, and chick posthatch growth to forty-two days. J. Appl. Poult. Res..

[bib0031] Van Roovert-Reijrink I.A.M., van der Pol C.W., Molenaar R., van den Brand H. (2018). Effect of warming profile at the onset of incubation on early embryonic mortality in long stored broiler eggs. Poult. Sci..

[bib0032] Wolanski N.J., Luiten E.J., Meijerhof R., Vereijken A.L.J. (2004). Yolk utilisation and chick length as parameters for embryo development. Avian Poult. Biol. Rev..

[bib0033] Wolanski N.J., Renema R.A., Robinson F.E., Carney V.L., Fancher B.I. (2006). Relationship between chick conformation and quality measures with early growth traits in males of eight selected pure or commercial broiler breeder strains. Poult. Sci..

[bib0034] Yassin H., Velthuis A.G.J., Boerjan M., van Riel J., Huirne R.B.M. (2008). Field study on broiler eggs hatchability. Poult. Sci..

[bib0035] Yoo B.H., Wientjes E. (1991). Rate of decline in hatchability with preincubation storage of chicken eggs depends on genetic strain. Br. Poult. Sci..

